# Methylenetetrahydrofolate Reductase (MTHFR) Gene Polymorphism and Infant’s Anthropometry at Birth

**DOI:** 10.3390/nu13030831

**Published:** 2021-03-03

**Authors:** Sofía Aguilar-Lacasaña, Inmaculada López-Flores, Beatriz González-Alzaga, María José Giménez-Asensio, F. David Carmona, Antonio F. Hernández, María Felicidad López Gallego, Desirée Romero-Molina, Marina Lacasaña

**Affiliations:** 1 Barcelona Biomedical Research Park (PRBB), Research Institute for Global Health (ISGlobal), Doctor Aiguader, 88, Barcelona, 08003 Barcelona, Spain; 2 Department of Genetics, Faculty of Sciences, University of Granada, Fuente Nueva s/n, 18071 Granada, Spain; dcarmona@ugr.es; 3 Instituto de Investigación Biosanitaria (ibs.GRANADA), Av. de las Fuerzas Armadas, 2, 18014 Granada, Spain; beatriz.gonzalez.easp@juntadeandalucia.es (B.G.-A.); mariajoseases@hotmail.com (M.J.G.-A.); ajerez@ugr.es (A.F.H.); 4 Andalusian School of Public Health (EASP), 18011 Granada, Spain; 5 Campus Universitario de la Cartuja, Cuesta del Observatorio, 4, 18011 Granada, Spain; 6 Andalusian Public Foundation for the Biosanitary Research of Eastern Andalusia-Alejandro Otero (FIBAO), Licinio de la Fuente Building, Dr. Azpitarte, 4, 18012 Granada, Spain; 7 Department of Legal Medicine and Toxicology, University of Granada School of Medicine, Health Sciences Technological Park, Avenida de la Investigación, 11, 18016 Granada, Spain; 8 Gynecology and Obstetrics Care Unit, Poniente Hospital, Ctra. de Almerimar, 04700 El Ejido-Almería, Spain; mfelopez@hotmail.com; 9 Statistics and Operations Research Department, Faculty of Sciences, University of Granada, Fuente Nueva s/n, 18071 Granada, Spain; 10 CIBER Epidemiology and Public Health (CIBERESP), Instituto de Salud Carlos III, Monforte de Lemos 3-5, Pabellón 11, Planta 0, 28029 Madrid, Spain

**Keywords:** MTHFR(677)C>T polymorphism, folate, folic acid, anthropometry at birth

## Abstract

Identification of causal factors that influence fetal growth and anthropometry at birth is of great importance as they provide information about increased risk of disease throughout life. The association between maternal genetic polymorphism MTHFR(677)C>T and anthropometry at birth has been widely studied because of its key role in the one-carbon cycle. MTHFR(677) CT and TT genotypes have been associated with a greater risk of low birth weight, especially in case of deficient intake of folic acid during pregnancy. This study aimed to analyze the association between the maternal MTHFR(677)C>T genetic polymorphism and anthropometry at birth in a population with adequate folate consumption. We included 694 mother–newborn pairs from a prospective population-based birth cohort in Spain, in the Genetics, Early life enviroNmental Exposures and Infant Development in Andalusia (GENEIDA) project. Women were genotyped for MTHFR(677)C>T SNP by Q-PCR using TaqMan© probes. Relevant maternal and newborn information was obtained from structured questionnaires and medical records. Results showed that maternal MTHFR(677)C>T genotype was associated with newborn anthropometry. Genotypes CT or CT/TT showed statistically significant associations with increased or decreased risk of large-for-gestational-age (LGA) or small-for-gestational-age (SGA) based on weight and height, depending on the newborn’s sex, as well as with SGA in premature neonates. The relationships between this maternal genotype and anthropometry at birth remained despite an adequate maternal folate intake.

## 1. Introduction

Anthropometric measurements of infants not only allow for assessing intrauterine growth, but also help to identify infants with a greater risk of suffering developmental disorders or diseases in adult life. Fetal ultrasound biometry measurement as well as birth anthropometry parameters (weight, height, chest circumference and head circumference adjusted for gestational age) are used as indicators to evaluate intrauterine fetal growth [1]. The terms small for gestational age (SGA) and large for gestational age (LGA) are defined as a function of whether anthropometric variables are below 10th percentile or above 90th percentile for gestational age, respectively. Infants born SGA or LGA have been associated with a significant perinatal morbidity and increased risk of diseases later in life as compared to infants born adequate for gestational age (AGA) [2,3,4,5].

Fetal growth and SGA or LGA results are influenced by different factors including maternal socio-demographic characteristics, health status, nutritional intake, environmental exposures, genetic background and gene-environment interactions [6,7,8].

Among nutrients, folate stands out for its role in fetal growth and placental development. Folate is the natural occurring state of folic acid (synthetic form) or vitamin B9, an essential water-soluble vitamin that must be obtained from diet. The main sources of folate are citrus fruits, legumes, green vegetables and liver. A daily dose of 400 micrograms of folic acid supplement is recommended to future mothers from the periconceptional period to prevent neural tube defects as well as low birth weight and preterm birth that are known to be associated to insufficient folate consumption during early pregnancy [9]. This supplementary dose is designed to achieve a daily folate intake of at least 600 micrograms per day during pregnancy. Folate is crucial during early pregnancy, where folate requirement increases 5 to 10 fold [10], as it participates in the one-carbon cycle, involved in synthesis of nucleic acids and aminoacids, cell division, tissue development and DNA methylation [11,12]. Moreover, as different studies point to potential adverse consequences of the intake of folic acid above a certain level, the recommended dose of folate intake during pregnancy is 600 to 1000 micrograms per day [5,13]. Vitamin B12 is also a relevant micronutrient, as it is cofactor for enzymes participating in the one-carbon cycle. A low level of maternal B12 together with increased level of homocysteine has been associated with impaired fetal growth, which suggests that an intake of methyl group donor nutrients such as folate is necessary in advanced stages of pregnancy [14]. The WHO recommended a B12 intake of 2.6 µg/day during pregnancy to support daily transfer to the fetus, although larger intakes (such as 1000 µg B12/day) are devoid of adverse effects [15].

On the other hand, alteration in folate levels can occur as a result of genetic poylmorphisms in gene coding for enzymes involved in its metabolism, which may lead to changes in their catalytic activity. One of these enzymes is methylenetetrahydrofolate reductase (MTHFR), which is often assessed in epidemiological studies due to the direct relationship between its activity and the levels of folate and also because of its implication in hyperhomocysteinemia [12,16]. Folate is converted by the MTHFR enzyme into the active form 5-methyltetrahydrofolate (5-MTHF), and carriers of the low activity allele have impaired lower ability to metabolize folate. Thus, the genetic polymorphism MTHFR(677)C>T (rs1801133) is responsible for folate availability, and decreased MTHFR enzymatic activity, together with certain environment conditions during pregnancy such as poor nutrition, low folate intake, hyperhomocysteinemia and active or passive exposure to tobacco smoke, have been associated with an increased risk of different adverse birth outcomes [17,18,19].

We hypothesized that key genetic polymorphisms responsible for folate availability influence fetal growth in populations with adequate folate intake. Therefore, the aim of the present study was to assess the association between the maternal MTHFR(677)C>T genetic polymorphism and anthropometry at birth in the Genetics, Early life enviroNmental Exposures and Infant Development in Andalusia (GENEIDA) birth cohort. This consists of a Mediterranean population of pregnant women supplemented with folic acid that reached the recommended folate dose during pregnancy.

## 2. Materials and Methods

### 2.1. Study Population and Design

This study was conducted on 694 mother/child pairs from the GENEIDA birth cohort. Pregnant women were recruited in the “Hospital El Poniente” in El Ejido, Almería (Southeast Spain) during the period from April 2014 to November 2017. Women were recruited in the first trimester of pregnancy (12–13 weeks of gestation) and then followed-up in the third trimester of pregnancy, at delivery. The inclusion criteria were: 16 years old or older, intention of giving birth at the reference hospital mentioned above, singleton pregnancy, pregnancy without assisted reproductive technology, lack of any chronic disease or being under medical treatment and absence of any language barrier.

### 2.2. DNA Extraction and Methylenetetrahidropholate Reductase Single Nucleotide Polymorphism (SNP) MTHFR(677)C>T (rs1801133) Genotyping

Blood samples were collected in the second trimester of pregnancy. Each sample (4 mL) was centrifuged at 2500 rpm for 15 min and the buffy coat (containing white blood cells) was carefully removed and stored at −20 °C until analysis. DNA was extracted using the “X-DNA Extraction Kit (XtremBiotech SL, Granada, Spain) following the manufacturer’s instructions. DNA integrity was checked by gel agarose gel electrophoresis. Quantification of DNA was carried out by spectrophotometry using the NanoPhotometerTM P-Class (IMPLEN, Schatzbogen, Germany). A total of 20 ng of dried-down genomic DNA was used for genotyping. Specific TaqMan^®^ Predesigned SNP Genotyping Assays (Applied Biosystems, Foster City, CA, USA) was used for the detection of the specific MTHFR(677)C>T (rs1801133) SNP in a QuantStudio 6 Flex Real-Time PCR System. As quality control, each series of amplifications included three negative controls without the DNA template to test for DNA contamination and three previously genotyped samples to test genotyping consistency.

### 2.3. Anthropometric Characteristics and Gestational Age

Gestational age and anthropometric measurements (weight, height, head circumference and chest circumference) at birth were obtained from medical hospital records. Weight index was calculated as the ratio of birth weight (grams) to height (cm^3^) [20].

Size for gestational age was estimated based on Carrascosa et al. 2004 [21] using neonatal gestational age at delivery, anthropometric measurements (AM: weight, height, head circumference) and sex. Newborns were categorized into three groups: SGA (AM less than 10th percentile for gestational age), AGA (AM 10th to 90th percentile for gestational age; which was the reference group), and LGA (AM greater than 90th percentile for gestational age).

A suitable alternative to identify SGA was using a chest circumference ≤ 30 cm as cut-off point.

### 2.4. Covariates

Information on covariates was obtained from questionnaires administered by personal interviews in the first and third trimester of pregnancy, and medical records. Covariates were selected according to clinical relevance and literature review of previous birth cohort studies in which association between genetic polymorphisms and birth outcomes was analyzed.

#### 2.4.1. Dietary Vitamin B12, Folate Intake and Folic Acid Supplementation Use

Information on diet was obtained using an adapted version of the food consumption of the Andalucian population based on a previously validated food frequency questionnaire (FFQ) [22,23]. In the first (12–13 weeks) and third trimester (36 weeks) of pregnancy, women were asked to report about their usual food consumption. The information obtained in the first trimester included intakes from 6 months pre-pregnancy until 12–13 weeks of gestation and the information obtained in the third trimester covered intakes between 14 and 36 gestational weeks. The FFQ includes the frequency of consumption and portion sizes for 119 food items, beverages including alcohol drinks, and intake of dietary supplements, including folic acid and other vitamins. The FFQ had nine options according to individual frequency consumption of each food or drink item. The i-Diet software (i-Diet; i-diet.es, Oviedo, Spain) [24] was used to estimate energy and daily nutrients intake (folate and B12 vitamins among others). Information on supplement intake included brand name, dosage per day and the start and end dates of consumption. This information was used to determine supplemental folate dose per day for each woman. Thereafter, data on background intake of folate from food was estimated according to its bioavailability in comparison with synthetic folic acid intake from food supplements. Thus, dietary folate was defined as 60% of the reported folate intake from foods [6,15,25,26]. Accordingly, the total folate intake in each trimester was calculated as supplemental folic acid +0.6 * folate intake from food [6]. Total B12 vitamin intake was calculated by the sum of the amount ingested from food and that from supplements.

A reproducibility and validation study of the FFQ was developed within the same population of pregnant women. The reproducibility analysis was conducted in a sample of 690 pregnant women who provided food consumption data for the first and third trimester of pregnancy. The Spearman correlation coefficients for folate and vitamin B12 intake estimated by the two FFQs were 0.486 and 0.385 *p* < 0.001, respectively. Validation analysis of the questionnaires was also carried out in a selected subsample of women (n = 43) who filled-in three 24-h dietary recalls in addition to the FFQs. The limits of agreement (LoA) for folate and vitamin B12 were 90.70% and 93.02%, respectively.

#### 2.4.2. Sociodemographic, Lifestyle and Obstetrics Variables

Maternal covariates were obtained from hospital medical records (gestational diabetes and hypertension) and questionnaires administered by trained interviewers in first and third trimester of pregnancy. These covariates were maternal height, self-reported pre-pregnancy weight and weight at week 36 of pregnancy, which allowed for calculation of weight gain during pregnancy and body mass index (BMI). Data collected on sociodemographic characteristics included maternal age, maternal education level, employment data, family income, marital status, parity, planned pregnancy, and lifestyles (tobacco smoking and alcohol consumption) during the pregnancy.

### 2.5. Statistical Analyses

The R Project for Statistical Computing (version 4.0.2, R Foundation for Statistical Computing, Vienna, Austria) was used for imputation of missing values of covariates. Multivariate imputations by chained equation in R (MICE package) were used to replace missing information [27]. Ten imputed datasets were generated; the mode was selected for non-continuous variables and the mean for the remaining variables.

Statistical analyses were performed using the IBM SPSS Statistics v.21 software. General characteristics of the study population were described using geometric mean, arithmetic mean, standard deviation and 95% confidence interval for continuous variables and absolute (n value) and relative frequencies (percentage) for categorical variables. Analyses were stratified by newborn sex.

The Student’s t test was used to compare means of continuous variables with a normal distribution. The non-parametric Mann–Whitney test was used to compare quantitative variables that failed to fit a normal distribution. The Chi-squared test was used for qualitative variables comparison (including differences in categorical variables by newborn sex) and also to examine whether genotype distribution was in Hardy–Weinberg equilibrium.

Bivariate and multinominal logistic regression analyses were used to evaluate association (odds ratio, OR) between MTHFR(677)C>T maternal genotypes and anthropometric variables for gestational age (SGA versus AGA and LGA versus AGA). Homozygous CC was the reference genotype used. OR greater than 1 was considered a positive association (risk effect) whereas OR values less than 1 indicated negative association (protective effect). All models were stratified by newborn sex.

Statistical significance was assumed for *p*-values < 0.1.

## 3. Results

The distribution of maternal genotype and allele frequencies is shown in Table 1. No significant differences were found when comparing by newborn sex. When observed frequencies were compared to the expected frequencies, no significant deviations from the Hardy–Weinberg equilibrium (HWE) were observed (*p* > 0.1).

The main clinical and sociodemographic characteristics of the study population are shown in Table 2 and Table 3. Table 2 summarizes information on continuous variables of newborns and their mothers. Significant differences were found for newborn weight, height, head circumference and chest circumference when compared by sex (*p* < 0.01). The remaining newborn anthropometric measures failed to show significant differences. Mean total folate and vitamin B12 intakes of pregnant women were within the recommended range during pregnancy. When compared by sex, mean maternal vitamin B12 intake during the first trimester of gestation was significantly lower for female newborn infants (*p* = 0.055).

Table 3 shows the general characteristics of newborns and women (categorical variables). Statistically significant differences by sex were observed only when newborns were classified as SGA, AGA or LGA based on height. The remaining anthropometric measures failed to show significant differences. About 80% of women reported alcohol consumption (any amount) at some point during their pregnancy, while a far lower percentage (around 16%) reported smoking during the pregnancy. Sixty-five percent and 77% of women had total folate intake over 600 µg, the recommended dose for the first and third trimester of pregnancy, respectively. Gestational diabetes was found to be more frequent in women who gave birth to male infants (*p* = 0.006).

Table 4 Mean anthropometric measures, weight, height, head circumference, chest circumference, weight index and gestational age, of male and female newborns did not show significant differences when compared by maternal MTHFR(677)C>T genotypes (Table 4).

The association between anthropometric measures of newborns (categorical variables) stratified by sex and maternal MTHFR(677)C>T genotype is shown in Table 5. Infants born LGA according to weight were significantly associated with the maternal genotype CT or CT/TT in both sexes. While female infants whose mother carried a T allele had an increased risk of being LGA (OR = 2.09 CI 95% (1.00;4.36) *p* < 0.05) male infants born to mothers carrying the T allele had a protective effect against LGA condition (OR = 0.52 CI 95% (0.26;1.06) *p* < 0.1). The same was observed when maternal genotype was CT or TT for female (OR = 1.89 CI 95% (0.94;3.81) *p* < 0.1) and male infants (OR = 0.49 CI 95% (0.25;0.93) *p* < 0.05).

Maternal genotype TT was also a protective factor for being born LGA according to height, but only in female infants (OR = 0.36 CI 95% (0.15;0.89) *p* < 0.05).

Although male infants had an increased risk of being born SGA according to weight index when their mothers had the heterozygous CT genotype (*p* < 0.1; OR = 1.60 CI 95% (0.97;2.62)), although statistical significance did not remain in the adjusted model. In the case of female premature infants, an increased risk of being born SGA was found for the maternal CT genotype (OR = 5.01 CI 95% (0.85;29.59) *p* < 0.1) or CT/TT (OR = 4.32 CI 95% (0.79;23.58) *p* < 0.1).

Head and chest circumferences did not show significant associations with the maternal genotype (*p* > 0.1)

## 4. Discussion

In this study of pregnant women from Southern Spain with adequate folate nutritional status, the MTHFR(677)C>T genetic polymorphism was associated with infant anthropometry at birth and with a higher risk of SGA in female premature infants.

The frequency of maternal MTHFR 677T allele of our population (38%) was in agreement with those reported for other populations, which also found no differences in the distribution of genotypes and allele frequencies by sex [28]. Newborns only differed in anthropometric measures of weight, height, head and chest circumferences as expected according to sex.

The common MTHFR genetic polymorphism (677)C>T (rs1801133) leads to the synthesis of a thermolabile enzyme, which may lead to high levels of homocysteine in the blood (hyperhomocysteinemia) that have been considered a risk factor for several adverse outcomes at birth. Previous studies have shown that the cytosine (C) to thymine (T) transition at position 677 within exon 4 of the MTHFR gene, which results in an alanine to valine amino acid substitution in the protein, is responsible for reducing enzyme activity in the homozygous TT and heterozygouw CT allelic variants by 60% and 30% of the CC genotype, respectively [12,29]. This reduced catalytic activity of MTHFR declines the production of 5-MTHF from folate. 5-MTHF is the active and circulating form of folate, which is a methyl donor for the conversion of homocysteine to methionine [12,30,31]. When methionine is converted back to homocysteine, the methyl group released causes DNA methylation [12]. Vitamin B12 also plays an important role in this cycle, as it is a necessary cofactor in the enzymatic conversion of homocysteine to methionine [32,33]. Other B vitamins, which have not been as extensively studied such as B6, play an interesting role in the one-carbon cycle. It should be noted that there are innumerable interrelations between B vitamins and may be influenced by genetic determinants, including some polymorphisms such as the one mentioned in this study [34].

In the absence of adequate folate availability, methionine production decreased, homocysteine accumulates and methylation reactions are compromised. Thus, folate, as a donor of carbon units in the one-carbon cycle, participates in the synthesis of nucleic acids as well as in the methylation of DNA sequences, including key placental genes. Folic acid supplementation during early pregnancy is intended to prevent the possible pathological conditions that a folate deficiency could cause [35,36,37]. This deficiency might be due to a poor dietary intake of green vegetables, fruits, legumes and animal origin food products or to a decreased bioavailability of folate due to a genetic background, for example, carriers or the unfavorable genotypes MTHFR-677CT/TT who may need supplemental folate intake [24,38,39,40,41].

The association between the maternal MTHFR(677)C>T genetic polymorphism and adverse birth outcomes has been previously reported in different populations, especially in pregnant women with inadequate folate intake. A recent meta-analysis showed that the maternal MTHFR(677)-T allele, and specifically carriers of the TT genotype, were associated with an increased risk of preterm birth and with low birth weight under most of the genetic models [18]. That study concluded that screening for MTHFR(677)C>T genetic polymorphism might play a key role for prevention of preterm birth and low birth weight in high risk pregnant women, especially with insufficient folate intake [18].

In our population, only 1% of pregnant women did not intake a folic acid supplements before or during the first trimester of pregnancy. The vast majority (99%) of pregnant women received a supplement of 400 or 500 micrograms of folic acid per day, which in almost all cases also included 2 to 2.6 micrograms of vitamin B12. The same folate intake was maintained during the third trimester of pregnancy. Thereby, the mean total folate intake was considered adequate, as it falls within the recommended dose of 600–1000 micrograms per day (679.79 µg/day (95% CI 669.78–689.80) during first trimester and 655.30 µg/day 95% CI 643.27–667.33 during the third trimester). This means that 77 and 65% of women had total folate intakes over the minimum recommended dose during the first and third trimester, respectively. Vitamin B12 total intake was always above the recommended dose of 2.6 µg/day during pregnancy. A daily folic acid intake of 400 µg per day has been reported to be sufficient to override the effects of unfavourable MTHFR genotypes on homocysteine and plasma folate levels [42]. This may be the case in our population, considering folic acid supplementation and background folate intake data, as well as high Vitamin B12 consumption. Despite this adequate folate status maintained throughout pregnancy we observed an association between heterozygous or homozygous carriers of the MTHFR(677)T variant and SGA and LGA categories according to weight, height and preterm birth. It appears that unfavorable MTHFR(677) genotypes require additional factors to overcome the metabolic defects caused by low MTHFR activity, otherwise the genetic factor by itself would impair fetal growth. An experimental study showed that folate supplementation in a cell line with low MTHFR activity did not significantly increase the concentration of 5-MTHF, the biologically active folate, when compared with normal MTHFR activity cells [43]. Thus, the metabolic defects caused by genetic polymorphisms of the gene encoding the MTHFR enzyme may require supplemental 5-MTHF to overcome such defects [43]. Moreover, previous studies have demonstrated the excellent safety profile for the Calcium L-5-methyltetrahydrofolic acid (L-5-MTHF-Ca). Thus, it would be interesting to assess in future studies a supplementation with L-5-MTHF-Ca as a safe alternative to 5-MTHF as a source of folate, in particular for pregnant women carrying the MTHFR(677)T variant [44].

Altered methylation pattern in placental genes involved in fetal growth could be one of the biological mechanisms that would explain the association between the MTHFR(677)C>T genetic polymorphism and anthropometric measures at birth. Hypomethylation of DNA sequences of that gene, which usually are imprinted, could lead to overgrowth. That is, placental hypomethylation may indiscriminately activate various imprinted and not imprinted genes, including those involved in fetal growth. Likewise, in utero exposure to adverse environmental conditions might impair DNA methylation through altered availability of methyl donors provided by the one-carbon pathway and this effect could be sex-specific. Drake et al., observed that maternal smoking was associated with sex-specific alterations in fetal liver vitamin B12, plasma homocysteine and expression of enzymes in the one-carbon cycle in fetal liver [45]. These mechanisms could in part explain the sex specific differences observed in our study, where mothers having a CT or CT/TT genotype had an increased risk of having an LGA female infant (according to weight) while it had a protective effect for LGA male infants.

Alterations in DNA methylation have been proposed as the possible link between MTHFR(677) unfavorable variant and increased risk of pregnancy complications Del Gobblo et al. [46]. That study did not find evidence for an association of the MTHFR(677)C>T polymorphism with altered placental DNA methylation using both genome-wide and site-specific approaches, but described a tendency for increased MTHFR(677)TT genotype in placentas from pregnancies complicated by pathologies such as preeclampsia and intrauterine growth restriction [45]. The inconsistencies in the results reported by different authors reveal that the effect of the MTHFR(677) polymorphism on DNA methylation is complex, and may likely be influenced by other polymorphisms in genes involved in the one-carbon cycle, and by the complex interactions between all them and also with an intrauterine adverse environment.

The current study has a number of strengths and limitations. The major limitation was the semi-quantitative assessment of the maternal dietary folate intake obtained by questionnaires. We do not have data on biomarkers in biological samples, which otherwise is seldom used in cohort studies in which information of food and vitamins consumption is commonly obtained through food frequency questionnaires. The questionnaire we used was adapted from one previously validated in a Mediterranean population of pregnant women. In this validation study significant correlations were observed between total folate and vitamin B12 intake estimated by FFQ and plasma folate and vitamin B12 concentrations (r = 0.53, *p* < 0.01 and r = 0.12, *p* < 0.05, respectively), and a percentage of agreement of 72% and 55%, respectively [23], which ensures the suitability of its use. Moreover, most of the study population was made up of Spaniards, which limits generalization of results across ethnicities. In any case, this homogeneity avoids genetic stratification due to geographical origin. Another limitation of this study is the lack of data on fetal MTHFR(677)C>T genotype. Because of the interplay of maternal and fetal genotypes, the missing fetal data prevent us from assessing the relative fetal contribution to the observed effect.

## 5. Conclusions

This study showed that maternal MTHFR(677)C>T genetic polymorphisms is associated with infant’s anthropometry at birth. Considering the potential effects of low folate availability due to diet and/or genetic background, it is necessary to continue emphasizing the importance of following the dietary recommendations during pregnancy, or at least to inform pregnant women on the risk of not following such recommendations. It is crucial to raise awareness on this issue, even in populations where folic acid supplementation has been implemented for years.

## Figures and Tables

**Table 1:** Genotypic and allele frequencies for the MTHFR(677)C>T (rs1801133) polymorphism. Frequencies are shown for the whole population and stratified by newborn sex.

Maternal Polymorphism (ID dbSNP)	Location (GRCh38) and Result	Genotypic and Allele Frequencies		
		Total (*n* = 694)	Male (*n* = 355)	Female (*n* = 339)
**MTHFR(677)C>T (rs1801133)**	Chr.1: 11796321 Ala→Val amino acid residue 222	CC = 0.39	CC = 0.40	CC = 0.38
		CT = 0.45	CT = 0.45	CT = 0.46
		TT = 0.16	TT = 0.15	TT = 0.16
		C = 0.62	C = 0.63	C = 0.61
		T = 0.38	T = 0.37	T = 0.39

**Table 2:** Clinical and demographic characteristics of the study population (continuous variables).

Characteristic		TOTAL (*n* = 596)		Male Infants (*n* = 355)		Female Infants (*n* = 339)		
		Arithmetic Mean (SD)	Geometric Mean	Arithmetic Mean (SD)	Geometric Mean	Arithmetic Mean (SD)	Geometric Mean	*p*-Value ^1^
**Newborn**	**Weight (g)**	3310.06 (451.41)	3277.10	3385.55 (458.05)	3352.95	3231.02 (431.04)	3199.50	<0.001
	**Height (cm)**	50.62 (2.14)	50.57	50.99 (2.18)	50.94	50.23 (2.28)	50.17	<0.001
	**Head circumference (cm)**	33.73 (1.57)	33.69	33.99 (1.54)	33.96	33.45 (1.55)	33.42	<0.001
	**Chest circumference (cm)**	32.96 (1.82)	32.91	33.19 (1.83)	33.14	32.72 (1.77)	32.67	0.001
	**Weight index (g/cm^3^)**	2.55 (0.25)	2.53	2.54 (0.25)	2.54	2.54 (0.25)	2.53	0.829
	**Gestational age (weeks)**	39.55 (1.45)	39.52	39.57 (1.46)	39.54	39.52 (1.45)	39.49	0.566
**Maternal**	**Age (years)**	31.26 (4.73)	30.89	31.39 (4.74)	31.02	31.12 (4.73)	30.74	0.496
	**Folate intake (μg/day)** **Pre-pregnancy and 1st Trimester ^2^**	679.79 (134.32)	664.79	686.27 (136.64)	671.21	673.01 (131.71)	658.13	0.153
	**Folate intake (μg/day)** **2nd y 3rd Trimester ^2^**	655.30 (161.40)	634.71	661.10 (166.79)	640.93	649.23 (155.57)	628.27	0.288
	**Vitamin B12 intake (μg/day)** **Pre-pregnancy and 1st Trimester**	10.50 (6.60)	9.37	10.93 (7.92)	9.63	10.04 (5.36)	9.11	0.055
	**Vitamin B12 intake (μg/day)** **2nd y 3rd Trimester**	9.91 (8.16)	8.38	9.74 (7.92)	8.30	10.09 (8.42)	8.47	0.886
	**Weight gain (g)**	11.20 (5.32)	*	11.21 (5.76)	*	11.20 (4.85)	*	0.706
	**BMI Prepregnancy**	24.34 (4.75)	24.02	24.28 (4.34)	24.10	24.38 (5.14)	23.93	0.671
	**BMI 3rd Trimester**	28.53 (4.61)	28.18	28.46 (4.20)	28.17	28.58 (5.01)	28.20	0.533

* Cannot be calculated due to negative values; ^1^ Student t-test was used for weight and weight index, (normal distributed variables), whereas the Mann–Whitney test was used for the remaining anthropometric measures (not normally distributed variables); ^2^ Total folate intake (calculated as supplemental folic acid + 0.6 * folate intake from food).

**Table 3:** Clinical and demographic characteristics of the study population (categorical variables).

Characteristic		Total (*n* = 694)	Male Infants (*n* = 355)	Female Infants (*n* = 339)	*p*-Value ^1^
		*n* (%)	*n* (%)	*n* (%)	
**Newborn**	Weight				
	SGA	36 (5.2)	19 (5.4)	17 (5.0)	0.782
	Normal	568 (81.8)	293 (82.5)	275 (81.1)	
	LGA	90 (13.0)	43 (12.1)	47 (13.9)	
	Height				
	SGA	33 (4.8)	13 (3.7)	20 (5.9)	0.079
	Normal	503 (72.5)	270 (76.1)	233 (68.7)	
	LGA	158 (22.8)	72 (20.3)	86 (25.4)	
	Head circumference				
	SGA	68 (9.8)	29 (8.2)	39 (11.5)	0.315
	Normal	621 (89.5)	323 (91.0)	298 (87.9)	
	LGA	5 (0.7)	3 (0.8) v	2 (0.6)	
	Chest circumference				
	SGA	24 (3.5)	12 (3.4)	12 (3.5)	0.908
	Normal	670 (96.5)	343 (96.6)	327 (96.5)	
	Weight index				
	SGA	220 (31.7)	116 (32.7)	104 (30.7)	0.799
	Normal	448 (64.6)	225 (63.4)	223 (65.8)	
	LGA	26 (3.7)	14 (3.9)	12 (3.5)	
	Prematurity				
	Yes	26 (3.7)	14 (3.9)	12 (3.5)	0.779
	No	668 (96.3)	341 (96.1)	327 (96.5)	
**Maternal**	Nationality				
	Spanish	618 (89.0)	320 (90.1)	298 (87.9)	0.395
	South American	8 (1.2)	5 (1.4)	3 (0.9)	
	European (not Spanish)	68 (9.8)	30 (8.5)	38 (11.2)	
	Education level				
	No studies	38 (5.5)	18 (5.1)	20 (5.9)	0.898
	Primary school	303 (43.7)	154 (43.4)	149 (44.0)	
	Secondary school	161 (23.2)	86 (54.2)	75 (22.1)	
	University studies	192 (27.7)	97 (27.3)	95 (28.0)	
	Smoking during pregnancy				
	Yes	116 (16.7)	61 (17.2)	55 (16.2)	0.735
	No	578 (83.3)	294 (82.8)	284 (83.8)	
	Alcohol consumption during pregnancy				
	Yes	564 (81.3)	281 (79.2)	283 (83.5)	0.144
	No	130 (18.7)	74 (20.8)	56 (16.5)	
	Folate intake pre-pregnancy and 1st Trimester ^2^				
	<600 μg/day	162 (23.3)	75 (21.1)	87 (25.7)	0.158
	≥600 μg/day	532 (76.7)	280 (78.9)	252 (74.3)	
	Folate intake 2nd and 3rd Trimester ^2^				
	<600 μg/day	243 (35.0)	124 (34.9)	119 (35.1)	0.962
	≥600 μg/day	451 (65.0)	231 (65.1)	220 (64.9)	
	Gestational diabetes				
	Yes	37 (5.3)	27 (7.6)	10 (2.9)	0.006
	No	657 (94.7)	328 (92.4)	329 (97.1)	
	Hypertension				
	Yes	14 (2.0)	8 (2.3)	6 (1.8)	0.651
	No	680 (98.0)	347 (97.7)	333 (98.2)	

^1^ Chi-squared test. ^2^ Total folate intake (calculated as supplemental folic acid + 0.6 * folate intake from foods).

**Table 4:** Differences in arithmetic and geometric means of anthropometric measurements (stratified by sex) according to the maternal genetic polymorphism of MTHFR(677)C>T.

		Male Infants (*n* = 355)				Female Infants (*n* = 339)			
		Maternal MTHFR(677)C>T Genotype				Maternal MTHFR(677)C>T Genotype			
		CC	CT	TT	*p*-Value	CC	CT	TT	*p*-Value
Weight (g)	AM	3402.69	3376.20	3367.50	0.840	3259.27	3214.58	3210.27	0.636
	(SD)	(482.26)	(457.95)	(394.47)		(413.74)	(449.90)	(421.19)	
	GM	3365.69	3344.17	3345.03		3229.50	3181.07	3180.76	
Height(cm)	AM	50.91	50.97	51.2	0.436	50.26	50.25	50.07	0.800
	(SD)	(2.32)	(2.10)	(2.04)		(2.25)	(2.39)	(2.08)	
	GM	50.86	50.93	51.22		50.21	50.20	50.03	
Head circumference (cm)	AM	34.02	34.02	33.83	0.597	33.60	33.33	33.44	0.301
	(SD)	(1.53)	(1.65)	(1.26)		(1.51)	(1.57)	(1.58)	
	GM	33.99	33.98	33.81		33.57	33.29	33.40	
Chest circumference (cm)	AM	33.20	33.16	33.25	0.795	32.82	32.64	32.73	0.602
	(SD)	(1.80)	(1.90)	(1.75)		(1.70)	(1.72)	(2.08)	
	GM	33.15	33.11	33.20		32.77	32.59	32.66	
Weight index (gr/cm^3^)	AM	2.57	2.54	2.51	0.242	2.56	2.53	2.55	0.681
	(SD)	(0.25)	(0.26)	(0.22)		(0.25)	(0.27)	(0.23)	
	GM	2.56	2.53	2.50		2.55	2.52	2.54	
Gestational age (weeks)	AM	39.49	39.65	39.57	0.687	39.63	39.44	39.45	0.423
	(SD)	(1.48)	(1.45)	(1.44)		(1.43)	(1.49)	(1.39)	
	GM	39.46	39.62	39.54		39.61	39.41	39.42	

Newborn anthropometric measures are presented as arithmetic mean (AM) and 95% confidence interval (CI 95%), and geometric mean (GM). Means for each genotype were compared by ANOVA in the case of weight and weight index (normally distributed variables) and by Kruskal–Wallis test for the remaining anthropometric measures (variables lacking a normal distribution).

**Table 5:** Associations between anthropometric measures at birth and maternal MTHFR(677)C>T genotype.

		Female Infants						Male Infants					
		SGA			LGA			SGA			LGA		
Characteristics	Genotype	SGA/AGA	OR Crude (IC 95%)	OR Adjusted (IC 95%)	LGA/AGA	OR Crude (IC 95%)	OR Adjusted (IC 95%)	SGA/AGA	OR Cruda (IC 95%)	OR Adjusted (IC 95%)	LGA/AGA	OR Crude (IC 95%)	OR Adjusted (IC 95%)
**Weight ^1^**													
	**CC**	4/112	Ref.	Ref.	14/112	Ref.	Ref.	8/111	Ref.	Ref.	24/111	Ref.	Ref.
	**CT**	11/117	2.63 (0.81;8.51)	2.78 (0.75;10.31)	26/117	1.78 (0.88;3.58)	2.09 ** (1.00;4.36)	9/134	0.93 (0.35;2.50)	0.87 (0.30;2.56)	15/134	0.52 * (0.26;1.03)	0.52 * (0.26;1.06)
	**TT**	2/46	1.22 (0.22;6.88)	1.43 (0.24;8.74)	7/46	1.22 (0.46;3.21)	1.41 (0.52;3.81)	2/48	0.58 (0.12;2.82)	0.66 (0.13;3.38)	4/48	0.39 * (0.13;1.17)	0.38 (0.12;1.17)
	**CT o TT**	13/163	2.23 (0.71;7.03)	2.38 (0.67;8.44)	33/163	1.62 (0.83;3.17)	1.89 * (0.94;3.81)	11/182	0.84 (0.33;2.15)	0.81 (0.29;2.26)	19/182	0.48 ** (0.25;0.92)	0.49 ** (0.25;0.93)
**Height ^2^**													
	**CC**	7/87	Ref.	Ref.	36/87	Ref.	Ref.	4/110	Ref.	Ref.	29/110	Ref.	Ref.
	**CT**	12/99	1.51 (0.57;4.00)	1.85 (0.63;5.45)	43/99	1.05 (0.62;1.78)	1.19 (0.69;2.05)	7/122	1.58 (0.45;5.54)	1.53 (0.26;8.91)	29/122	0.90 (0.51;1.60)	0.93 (0.52;1.66)
	**TT**	1/47	0.26 (0.03;2.21)	0.26 (0.03;2.37)	7/47	0.36 ** (0.15;0.87)	0.36 ** (0.15;0.89)	2/38	1.45 (0.26;8.22)	1.42 (0.40;5.10)	14/38	1.40 (0.67;2.92)	1.41 (0.67;2.96)
	**CT o TT**	13/146	1.11 (0.43;2.88)	1.26 (0.45;3.57)	50/146	0.83 (0.50;1.37)	0.91 (0.54;1.52)	9/160	1.55 (0.47;5.15)	1.45 (0.43;4.91)	43/160	1.02 (0.60;1.73)	1.04 (0.61;1.78)
**Head circumference ^3^**													
	**CC**	15/114	Ref.	Ref.	1/50	Ref.	Ref.	13/129	Ref.	Ref.	1/129	Ref.	Ref.
	**CT**	20/134	1.13 (0.56;2.32)	1.07 (0.51;2.25)	0/134	no value	no value	12/144	0.83 (0.36;1.88)	0.70 (0.30;1.63)	2/144	1.79 (0.16;19.9)	2.11 (0.18.24.53)
	**TT**	4/50	0.61 (0.19;1.93)	0.61 (0.19;1.97)	1/50	2.28 (0.14;37.18)	2.25 (0.13;38.13)	4/50	0.79 (0.25;2.55)	0.69 (0.21;2.30)	0/50	no value	no value
	**CT o TT**	24/184	0.99 (0.50;1.97)	0.95 (0.47;1.93)	1/184	0.62 (0.04;10.00)	0.59 (0.04;9.83)	16/194	0.82 (0.38;1.76)	0.70 (0.32:1.54)	2/194	1.33 (0.12;14.82)	1.61 (0.14;18.86)
**Chest circumference ^4^**													
	**CC**	4/126	Ref.	Ref.	-	-	-	4/139	Ref.	Ref.	-	-	-
	**CT**	4/150	0.84 (0.21;3.43)	0.50 (0.06;4.14)	-	-	-	6/152	1.37 (0.38;4.96)	3.54 (0.60;20.92)	-	-	-
	**TT**	4/51	2.47 (0.60;10.26)	3.29 (0.48;22.34)	-	-	-	2/52	1.34 (0.24;7.52)	2.26 (0.25;20.73)	-	-	-
	**CT o TT**	8/201	1.25 (0.37;4.25)	1.17 (0.21;6.47)	-	-	-	8/204	1.36 (0.40;4.61)	3.09 (0.58;16.56)	-	-	-
**Weight index ^5^**													
	**CC**	40/84	Ref.	Ref.	6/84	Ref.	Ref.	39/97	Ref.	Ref.	7/97	Ref.	Ref.
	**CT**	51/97	1.10 (0.67;1.83)	1.04 (0.62;1.75)	6/97	0.87 (0.27;2.79)	1.02 (0.31;3.42)	59/92	1.60 * (0.97;2.62)	1.48 (0.90;2.45)	7/192	1.05 (0.36;3.12)	0.85 (0.27;2.63)
	**TT**	13/42	0.65 (0.31;1.34)	0.61 (0.29;1.29)	0/42	no value	no value	18/36	1.24 (0.63;2.45)	1.14 (0.57;2.28)	0/36	no value	no value
	**CT o TT**	64/139	0.97 (0.60;1.56)	0.91 (0.56;1.49)	6/139	0.60 (0.19;1.94)	0.69 (0.21;2.28)	77/128	1.50 * (0.94;2.39)	1.39 (0.86;2.23)	7/128	0.76 (0.26;2.23)	0.60 (0.19;1.84)
**Prematurity ^6^**		**(yes/no)**						**(yes/no)**					
	**CC**	2/128	Ref.	Ref.	-	-	-	6/137	Ref.	Ref.	-	-	-
	**CT**	8/146	3.51 (0.73;16.82)	5.01 (0.85;29.59) *	-	-	-	6/152	0.90 (0.28;2.86)	0.81 (0.25;2.67)	-	-	-
	**TT**	2/53	2.42 (0.33;17.60)	3.03 (0.37;25.10)	-	-	-	2/52	0.88 (0.17;4.49)	0.97 (0.19;5.05)	-	-	-
	**CT o TT**	10/199	3.22 (0.69;14.92)	4.32 (0.79;23.58) *	-	-	-	8/204	0.89 (0.30;2.64)	0.85 (0.28;2.56)	-	-	-

* *p* < 0.1, ** *p* < 0.05. ^1^ Model adjusted for BMI at 36 weeks of gestation, hypertension, alcohol consumption and tobacco smoking during pregnancy. ^2^ Model adjusted for BMI at 36 weeks of gestation, hypertension and maternal age. ^3^ Model adjusted for maternal age, recommended folate intake in 2nd and 3rd trimester of pregnancy and tobacco smoking during pregnancy. ^4^ Model adjusted for hypertension, alcohol consumption during pregnancy and vitamin B12 intake in 2nd and 3rd trimester of pregnancy. ^5^ Model adjusted for BMI at 36 weeks of gestation, tobacco smoking during pregnancy and vitamin B12 intake in 2nd and 3rd trimester of pregnancy. ^6^ Model adjusted by hypertension and tobacco smoking during pregnancy.
